# Postprandial Peaking and Plateauing of Triglycerides and VLDL in Patients with Underlying Cardiovascular Diseases Despite Treatment

**DOI:** 10.5812/ijem.4783

**Published:** 2012-09-30

**Authors:** Clarissa E. Samson, Ana Lyza B. Galia, Khristine Ivy C. Llave, Manuel B. Zacarias, Leilani B. Mercado-Asis

**Affiliations:** 1 Section of Endocrinology and Metabolism, University Of Santo Tomas Hospital, Manila, Philippines; 2 Dietary Services, University of Santo Tomas Hospital, Manila, Philippines; 3 Section of Cardiology, University of Santo Tomas Hospital, Manila, Philippines

**Keywords:** Postprandial Lipemia, Cardiovasular Disease, Triglyceride, VLDL

## Abstract

**Background:**

Dyslipidemia is associated with cardiovascular morbidities and mortality. Currently, fasting lipid profile determination is used to monitor treatment response. Recently, postprandial lipemia is of increasing interest because of its atherogenic and thrombogenic potential and also was found to be more predictive for cardiovascular diseases.

**Objectives:**

To demonstrate postprandial lipemia among patients with cardiovascular diseases despite low fat diet, normal fasting lipid profile and even statin regimen.

**Patients and Methods:**

Patients aged 40-80 years old with cardiovascular diseases (i.e. coronary artery disease and cerebrovascular disease) more than 6 months, on statin treatment for more than 6 months and normal fasting lipid profile (according to NCEP ATP III guidelines) were included.

Study exclusion criteria were pregnancy, acute cardiovascular events < 6 months, hepatic or renal failure. Finally, twelve patients were included.

**Results:**

The triglyceride level showed a significant rise from fasting to 2 hours after breakfast with a mean difference of 23.86 mg/dL (P =0.012). The level peaked at 4 hours after breakfast with a mean difference (MD) of 72.02 mg/dL (P =0.002). Subsequent triglyceride levels plateaued and were significantly higher than the baseline (P <0.05) until the 12th hour of observation. VLDL levels showed a similar pattern. Levels increased significantly from fasting to 2h after breakfast (mean difference: 4.49 mg/dL, P = 0.007), then plateaued and further increased 4 hours after breakfast (MD: 14.01 mg/dL, P = 0.002). VLDL levels were significantly higher than fasting (P < 0.05) and did not return to baseline until the 12th hour of observation. In contrast, the levels of total cholesterol, HDL and LDL decreased postprandially.

**Conclusions:**

Triglyceride and VLDL peaking and plateauing were observed in patients with cardiovascular diseases despite low fat diet, normal fasting lipid profile and statin regimen. These findings may raise more attentions in monitoring and management of dyslipidemia in patients with cardiovascular and cerebrovascular events.

## 1. Background

Dyslipidemia is one of the most prominent risk factors of atherosclerosis leading to cardiovascular diseases. In the Philippines, its incidence has significantly increased from 2003 to 2008 (1).

Atherosclerosis is initiated by vascular endothelium dysfunction followed by formation of macrophage foam cells, which is generated by scavenging of lipids from plasma lipoproteins. Accumulation of foam cells and then proliferation of vascular smooth muscle cells (VSMCs) causes the appearance of fatty streaks, the first visible lesions in the vessel wall (2).

Following food consumption, triglycerides are transported from the small intestines via chylomicrons through the bloodstream. Lipolysis of the triglycerides within chylomicrons, catalyzed by lipoprotein lipase in tissues, transforms these particles into atherogenic, triglyceride-rich remnant lipoproteins. These remnants are easily taken up by macrophages, in contrast to LDL, which need to be modified first. The macrophages change into highly atherogenic foam cells when lipid uptake exceeds lipid clearance (3, 4).

It has been established that fasting total plasma cholesterol and LDL cholesterol are the best biomarkers of plasma for prediction cardiovascular diseases (CVD) risk. With monitoring LDL levels, prevention of CVD is more applicable, which is performed mainly based on a pharmaceutical approach (statins prescription), which has been proved to be extremely effective (5). Despite the fact, LDL elevation is absent in many patients with atherosclerosis and about 1/3 of cardiac events remains to be unpredicted using this parameter. Furthermore, in fasting normolipidemic subjects, increased CVD risk is associated with an exaggerated postprandial lipemic response (6, 7). As early as the 1970s, Zilversmit has hypothesized that postprandial increase in lipoproteins, specifically, cholesterol laden chylomicrons, contributes greatly to the higher atherosclerotic process risk (7). Postprandial lipemia (PPL) defined as a rise in triglyceride-rich lipoproteins (TRLs), including chylomicron remnants (CMRs) and remnant lipoproteins (RLPs), after eating, has drawn an increasing interest recently because of its association with cardiovascular events. Chylomicron remnants (CMRs) have been shown to penetrate the artery wall and to be retained within the intima (6) and remnant-like lipoproteins (RLPs) have been found in human atherosclerotic plaque as well (8, 9). CMRs and TRLs have also been demonstrated to cause endothelial dysfunction, macrophage foam cell formation and the proliferation of VSMCs (10). This is evidenced by carotid intima thickening, decreased in bloodstream flow due to stricture caused by presence of PPL, which finally leads to arterial stiffness (11-14). It was also found that hemostatic variables like clotting factors (i.e. increased factor VII_a_ activity, procoagulant effect and increased levels of plasminogen activator inhibitortype 1 and anti-fibrinolytic effect), platelet reactivity and monocyte cytokine expression, may be increased with postprandial lipemia (9, 12, 15-17). Cohn showed in his study performed on atherogenicity capability of remnant lipoproteins that patients with coronary artery diseases often have higher postprandial lipid levels compared to healthy subjects (18). In another investigation performed by Lundman, patients with established coronary artery diseases (CAD) were shown to have a prolonged PPL level after a fatty meal (19). It’s a matter of fact that presence of abnormal PPL is associated with progression of CAD and carotid artery diseases.

A local study by So, et al. in 2003 demonstrated occurrence of PPL in healthy Filipino volunteers after a high fat meal of different concentrations (20). Moreover, in 2008, Gabriel et al. were able to show that sustained postprandial lipemia occurs in Filipino healthy volunteers after high fat meals given in 3 meals and 2 snacks (21).

## 2. Objectives

The diagnosis and management of dyslipidemia in patients with cardiovascular problems have been established based on fasting lipid profile. Our study aimed to determine if postprandial lipemia will persist despite administration of standardized low fat meals, statin regimen and normal fasting levels in high risk patients with established cardiovascular and cerebrovascular events.

## 3. Patients and Methods

This was a descriptive study of patients aged 40 to 80 years old who had cardiovascular disease, i.e. coronary artery disease or cerebrovascular disease/ stroke for more than 6 months and had been maintained on statin regimen for more than 6 months. These patients were recruited preliminary for screening fasting lipid profile, complete blood count (CBC), fasting blood sugar (FBS), creatinine, alanine aminotransferase (ALT) and total protein, albumin and globulin (TPAG). Only those patients with normal fasting lipid profile were included in the study. Normal fasting lipid profile is defined using the NCEP ATP III published guidelines for the diagnosis, evaluation, and treatment of high blood cholesterol levels in adults which include: Total cholesterol (TC) < 200 mg/dL, triglyceride (TG) < 150 mg/dL, and LDL cholesterol < 100 mg/dL (22). The patients with the following characteristics were excluded from the study: pregnancy, with acute cardiovascular events within the past 6 months and those with hepatic or renal failure. Renal impairment was defined as serum creatinine level greater than 1.5 mg/dL.

The study protocol was discussed with the recruited subjects in details. Then they were asked to sign informed consent forms. The study was conducted in accordance with the ethical principles laid down in the Declaration of Helsinki. The subjects were scheduled for a whole day meal and blood extraction. They were instructed not to eat anything overnight for 10 hours. On the day of examination, they stayed in a rented room near the UST hospital, where they were accompanied by a medical technologist, a research assistant and a physician (the author). The subjects were given standard Filipino low fat diet (30 kcal/kg) prepared by the UST Dietary Department, composed of 60% carbohydrate, 25% fat, and 15% protein, divided into 3 meals given at 0800H, 1200H, 1800H and 2 snacks given at 1000H and 1600H. The participants were not allowed to eat anything aside the prepared meals. Only water was allowed to be drunk by the patients. They were asked to refrain from smoking and any strenuous activity during the study. All medications being maintained by the patients (i.e. antihypertensive, antiplatelet, antidiabetic agents and statin) were allowed to be taken.

A cannula with a three-way stopcock was placed in the median cubital vein or any other accessible vein of each subject. Blood samples were taken for baseline measurement before breakfast (0800H), which represents the fasting determinations; and every two hours thereafter until the 14th hour of observation blood sampling were performed. The postprandial lipid determinations coincide with premeal/ presnack determination, 2hrs and 4hrs after each main meal and snacks. Blood samples were sent to a central laboratory for determination of lipid profile. The lipid levels were analyzed using the Hitachi 902 analyzer (specific assay used was ELISA) by a single medical technologist who was blinded to the patients profile.

We noted and analyzed the differences between baseline and postprandial TC, TG, HDL, LDL, and VLDL levels. Analysis of Variance (ANOVA) was used to analyze the differences amongst the baseline and postprandial lipid measurements. Further analysis was done using the post- hoc Wilcoxon signed rank test.

## 4. Results

A total of 12 patients aged 60 +/-9 were included finally. 10 were males and 2 were females. The patients BMI ranged from 20 to 26 kg/m^2^. Only two patients were classified as obese class 1 (based on the Asia Pacific classification of Obesity). Six were non smokers; six were smokers but have stopped smoking for more than 8 years. Seven had coronary artery diseases, five had cerebrovascular disease (i.e. stroke). All were maintained on their prescribed statin regimen (i.e atorvastatin, rosuvastatin or simvastatin). Six were diabetic but with good glycemic control at the time of study. The rest did not have diabetes mellitus (See Table 1).

**Table 1:** Summary of Patients Characteristics.

Patient	Age	BMI a	Statin Treatment and Dosage	CV Event	DM	Smoking Status
1	46	22.14	A a 80 mg	CAD a	none	stopped 2 years
2	52	20.1	S 80 mg	CAD	none	stopped 5 years
3	52	26.4	A 40mg	CAD	none	no
4	60	24.69	A 80 mg	CAD	none	no
5	67	24.2	A 10 mg	CAD	+	stopped 7 years
6	57	22.1	A 20 mg	CAD	+	stopped 4 years
7	75	19.8	A 40 mg	CVA	+	stopped 10 years
8	61	21.5	A 20 mg	CVA	+	no
9	75	20.6	A 40 mg	CAD	+	stopped > 20 years
10	57	25.67	R a 10 mg	CVA	+	no
11	63	21.4	S a 40 mg	CVA	none	no
12	58	22.6	A 20 mg	CVA	none	no

^a^Abbreviations: A, Atorvastatin; R, Rosuvastatin; S, Simvastatin; CAD, Coronary artery disease; CVD, Cerebrovascular disease

The postprandial lipid profile was taken every 2 hours after fasting determination. The difference of each postprandial lipid levels from the baseline/fasting was analyzed.

**Figure 1:**
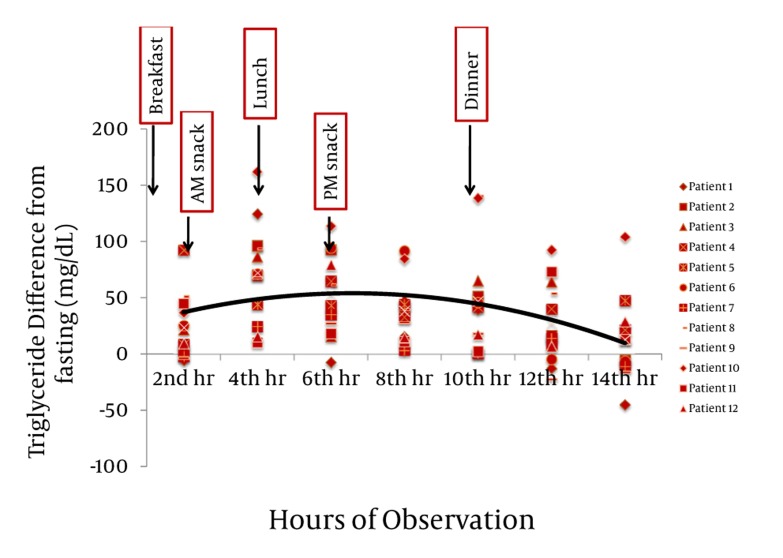
The Difference of Each of the Postprandial Triglyceride (TG) Levels From the Fasting TG Levels. The average of the differences is represented by the black line.

**Figure 2:**
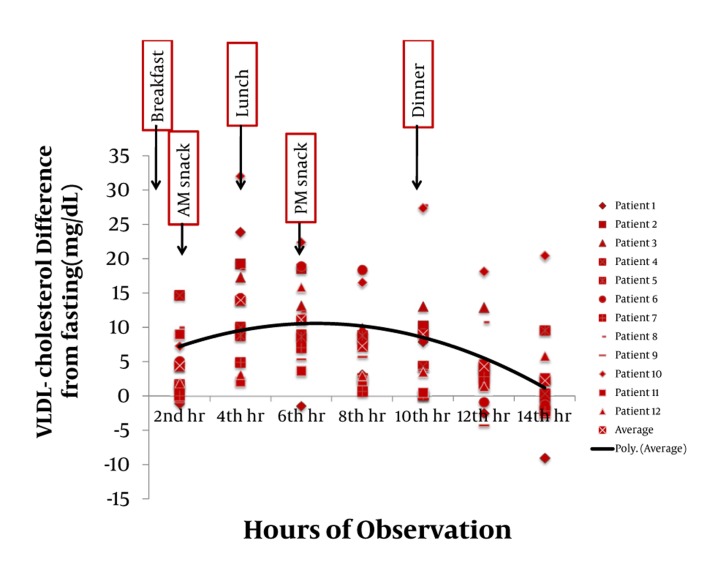
The Difference of Each of the Postprandial VLDL- Cholesterol Levels From the Fasting VLDL Levels. The average of differences is represented by the black line.

Majority of postprandial triglyceride levels were higher than fasting levels as reflected by the positive difference (see Figure 1). Statistical analysis of these postprandial differences from fasting triglyceride levels showed a significant rise from fasting to 2 hours after breakfast with a mean difference of 23.86 mg/dL (P = 0.012). The level sharply increased 4h after breakfast with a mean difference of 72.02 mg/dL (P = 0.002). Subsequent triglyceride levels plateaued and were significantly higher than the baseline (P < 0.05) until the 12th hour of observation (see Table 2, Figure 2). VLDL levels showed a similar pattern. Levels increased significantly from fasting to 2h after breakfast (MD: 4.49 mg/dL, P =0.007), then plateaued and further increased 4 hours after breakfast (MD: 14.01 mg/dL, P = 0.002). Subsequent VLDL levels were significantly higher than fasting (P < 0.05) and did not return to baseline until the 12^th^ hour of observation period (See Table 3, Figures 2 and 4). In contrast, the levels of Total Cholesterol, HDL and LDL were decreased postprandially (see Figures 5, 6 and 7, respectively).

**Table 2:** Difference of the Postprandial Triglyceride Levels From the Fasting Level.

	VLDL Difference From Fasting, mg/dL a	*P* value
2nd h	23.86	0.012
4th h	72.02	0.002
6th h	53.51	0.003
8th h	38.47	0.002
10th h	47.098	0.002
12th h	27.76	0.04
14th h	13.17	0.35

^a^The maximum difference was 72.02 mg/dL noted at the 4th hour of observation

**Table 3:** Difference of the Postprandial VLDL Levels From the Fasting Level.

	VLDL Difference From Fasting (mg/dL)	*P* value
2nd h	4.49	0.007
4th h	14.01^a^	0.002
6th h	11.15	0.003
8th h	7.31	0.002
10th h	9.04	0.002
12th h	4.36	0.041
14th h	2.27	0.35

^a^The Maximum Difference Was 14.01 mg/dL Noted at the 4th Hour of Observation.

**Figure 3:**
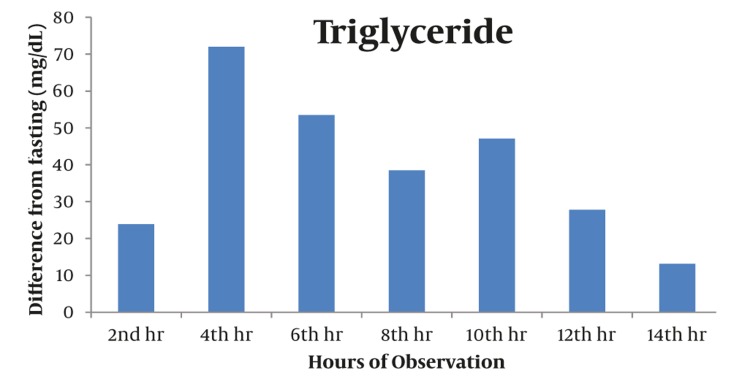
Triglyceride Levels Showed an Increasing Trend From The Baseline Until Its Peak at 4th Hour and Decreased Thereafter. All Values Were Significantly Elevated From Baseline Until the 12th Hour. P <0.05

**Figure 4:**
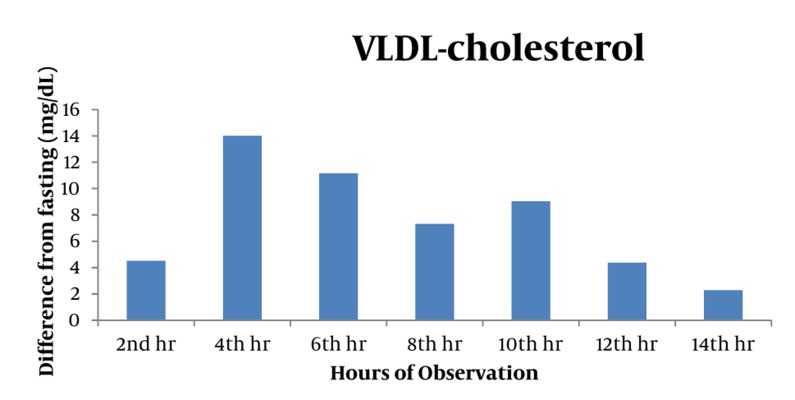
VLDL Levels Showed an Increasing Trend From the Baseline Until Its Peak at 4th Hour and Decreased Thereafter. All values were significantly elevated from baseline until the 12th hour. *P <0.05

**Figure 5:**
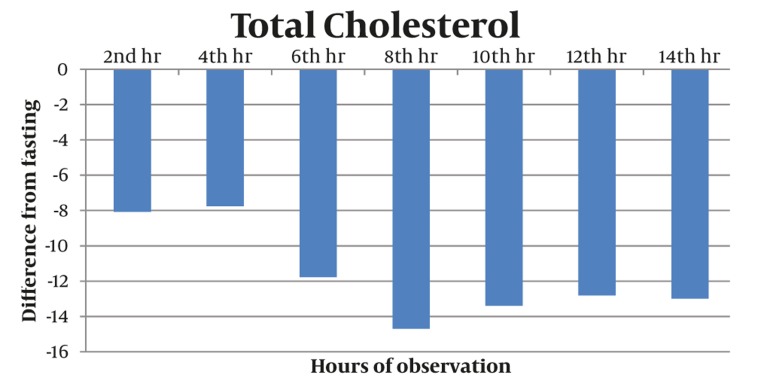
Postprandial Levels of Total Cholesterol Showed a Decreasing Trend as Shown by the Negative Differences From the Baseline. The lowest postprandial level occurred at the 8th hour with a maximum difference of (-) 14.69 mg/dL

**Figure 6:**
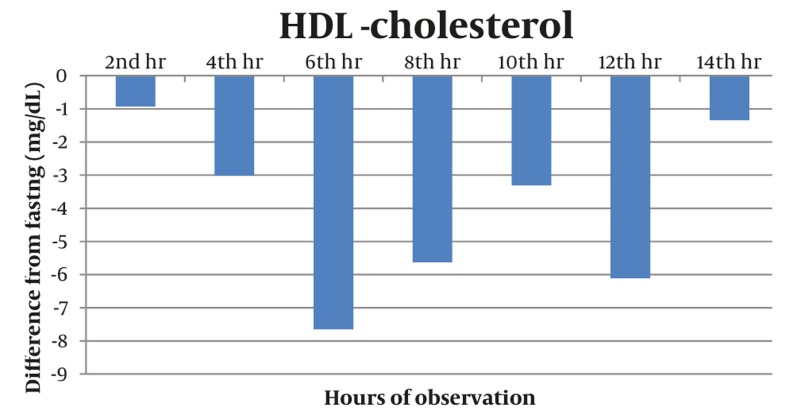
The HDL Levels Were Decreased Postprandially. The lowest postprandial level occurred at the 6th hour with a maximum difference from the fasting level of(-) 7.65 mg/dL.

**Figure 7:**
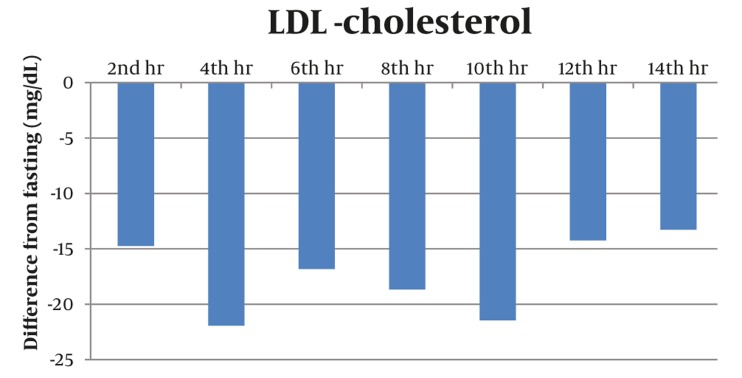
The Postprandial LDL Levels Were Decreased Postprandially With no Specific Pattern. The lowest postprandial level occurred at the 4th hour with a maximum difference from the fasting level of (-) 21.92 mg/dL.

## 5. Discussion 

The current management of dyslipidemia is low fat diet and statin regimen with the goal of reaching normal fasting lipid profile in order to circumvent cardiovascular events consequently. However, as mentioned, postprandial lipemia is associated with significant cardiovascular diseases risk.

Occurrence of postprandial lipemia has been demonstrated in healthy people in different studies. Locally in 2003, So et al. have characterized patterns of lipid profile changes postprandially in healthy Filipino volunteers after oral fat challenge test (OFCT) with varied percentages of fat content (40% oral fat challenge test (OFCT), 45% OFCT, 50% OFCT). The study showed that triglyceride levels have increased to peak levels up to 274 to 310 mg/dL at median time of 4 to 5 hours after a high fat meal. Additionally LDL showed a decreasing trend postprandially (20). The elevated postprandial triglyceride levels may reflect a delay in clearance of triglyceride-rich particles which can lead to further accumulation of these atherogenic particles (23).

In 2007, postprandial lipemia was proven to be an independent risk factor for cardiovascular events in a study of 1,793 adults from the Copenhagen general population study. The study showed that increasing levels of nonfasting triglyceride were associated with increased risk of MI, IHD, and consequently death in men and women. Their findings propose that every 1 mmol/L increase in non fasting triglyceride levels corresponds to further increase in the hazard ratio of the cardiovascular outcomes (24). In a prospective study by Bansal, et al. of 26, 509 women with median follow-up of 11.4 years, showed that cardiovascular events correlated more with nonfasting triglyceride values than fasting triglyceride independent from traditional cardiac factors, levels of other lipids and markers of insulin resistance (3).

The results of our study, involving a set of high risk patients with cardiovascular diseases who have normal fasting lipid profile and maintained on statin regimen, showed significant postprandial rise and plateauing of TG and VLDL levels starting 2 hours after fasting. These levels peaked at 4^th^ hour after the 1^st^ meal/breakfast and were maintained to be significantly higher than the baseline during 12 hours of observation.

In the analysis of our results, we opted to determine the incremental postprandial lipid concentration in relation to the baseline value, which was calculated by subtracting baseline values from those obtained postprandially. Bravo et al. recommended that because of current lack of reliable reference values available, it will be necessary, at least in the preliminary phase, to evaluate individual postprandial responses by determining the incremental postprandial TG concentration in relation to the baseline values. For follow-up, in the case of baseline and 4h determinations, the incremental concentrations obtained by subtracting baseline values from those obtained postprandially (baseline value becomes 0) may represent the best way to indicate the relative changes from baseline at different time points. These could be useful to the clinicians, not only to evaluate the postprandial response but also the way in which it may be influenced by changes in lifestyle habits or pharmaceutical therapy (10). Corollary to this, a study by Nordestgaard and Langstead in 2010 concluded that the maximum increase of triglyceride in individuals with and without diabetes is up to 17.6 mg/dL (0. 2 mmol/L) (19). Our results showed that 2 hours after breakfast, the triglyceride level increased by 23.86 mg/dL (0.27 mmol/L). The maximum increase was noted at the 4^th^ hour of observation at 72.02 mg/dL (0.82 mmol/L).

With regards to the timing of significant postprandial TG peak, our result is similar to a number of studies which showed that triglycerides and remnant lipoprotein concentrations both typically increase to their peaks after approximately 4 hours and decline thereafter in response to a meal. TG levels were taken 4 hrs after a meal has the greatest association with higher risks of cardiovascular events (3, 24).

Bravo et al. had established a reference interval values for nonfasting TG as follow (in mmol/L): healthy < 2.0; intermediate 2.1 to 2.7; altered > 2.8. However, subjects in the mentioned study did not have cardiovascular diseases and were given high fat meal. They also noted that these values need to be evaluated in individual populations (10).

The significant plateauing, or the sustained postprandial increase, of the postprandial TG and VLDL levels shown in this study are also of significant importance. All the postprandial TG values up to the 12^th^ hour of observation were significantly higher than the baseline at a range of 23.86 to 72.02 mg/dL (0.27 to 0.82 mmol/L). Thereby a similar pattern has been observed for VLDL.

Different studies have shown that long duration of PPL and repetition of meals during daytime leads to a hazardous postprandial lipemia in CVD patients (25-28). Such that prolonged lipemia leads to an increased opportunity for transfer of core lipids between the TRLs and HDL. This combination of high plasma TG concentrations, increased small dense LDL and low HDL are known as the lipid triad or atherogenic lipoprotein profile.

The postprandial VLDL levels showed a similar pattern to TG as mentioned above. However, there is still a lack of data regarding VLDL values. Although in our analysis, VLDL had a significant postprandial rise and is considered as a component of postprandial lipemia as well.

With our results and data of other studies presented, this postprandial phenomenon should be addressed especially in this set of high risk patients in order to avoid recurrence of cardiovascular events. Of note, Boccalondro et al, have shown that patients with coronary artery diseases have a prolonged postprandial lipemia compared to healthy individuals (29).

Thus, clinicians should be more precise in addressing postprandial lipemia in this set of patients. With the emerging evidences which indicate that postprandial dyslipidemia is a significant culprit in the development of atherosclerosis that may lead to an increased cardiovascular morbidities and mortality, a paradigm shift in the diagnosis and management of dyslipidemia seems to be inevitable. A random lipid level determination at clinic, preferably within 4 to 6 hours from the patient’s last meal, may serve as a more convenient and reliable monitoring scheme for patients compliance and medication adjustments.

A number of different pharmacological approaches may be effective in reducing postprandial hyperlipidemia in obese and diabetic patients, including statins, fenofibrate, nicotinic acid, and oral omega-3 fatty acid supplements (23, 30, 31). In the Philippines, different groups have demonstrated that orlistat may offset postprandial triglyceride and VLDL peaking and plateauing (21, 32, 33).

Patients with established cardiovascular and cerebrovascular events may need combination treatment of statins and fibrates with probable sequential addition of orlistat if needed following fatty meals.

In this study population, significant postprandial Triglyceride and VLDL peaking and plateauing were observed in patients diagnosed with cardiovascular diseases despite low fat diet, normal fasting lipid level and statin regimen. Decreasing levels in postprandial total cholesterol, HDL and LDL were observed.

These findings may lead to a paradigm shift in the monitoring/follow-up of dyslipidemia patients.

These may also be used in any future discussion in modification of the management of dyslipidemia, now addressing the postprandial lipemia.

We recommend that in future studies, a larger population size be recruited to represent the general population more accurately. A series of studies on this high risk patients that would document recurrence of CV events despite their compliance to the standard dyslipidemia treatment and normal fasting lipid profile, will further strengthen the importance of postprandial lipemia.
